# Interaction between *Pasteurella multocida* B:2 and its derivatives with bovine aortic endothelial cell (BAEC)

**DOI:** 10.1186/s12917-017-1109-1

**Published:** 2017-06-19

**Authors:** Nuriqmaliza M. Kamal, M. Zamri-Saad, Mas Jaffri Masarudin, Sarah Othman

**Affiliations:** 1 Department of Cell and Molecular Biology, Faculty of Biotechnology and Biomolecular Sciences, Universiti Putra Malaysia, 43400 UPM Serdang, Selangor Malaysia; 2 Research Centre for Ruminant Diseases, Faculty of Veterinary Medicine, Universiti Putra Malaysia, 43400 UPM Serdang, Selangor Malaysia; 3 Present address: Department of Cell and Molecular Biology, Faculty of Biotechnology and Biomolecular Sciences, Universiti Putra Malaysia, 43400 UPM Serdang, Selangor Malaysia

**Keywords:** *Pasteurella multocida*, Haemorrhagic septicaemia, Bovine aortic endothelial cell, Bactofection, DNA vaccine

## Abstract

**Background:**

*Pasteurella multocida* B:2 causes bovine haemorrhagic septicaemia (HS), leading to rapid fatalities in cattle and buffaloes. An attenuated derivative of *P. multocida* B:2 GDH7, was previously constructed through mutation of the *gdhA* gene and proved to be an effective live attenuated vaccine for HS. Currently, only two potential live attenuated vaccine candidates for HS are being reported; *P. multocida* B:2 GDH7 and *P. multocida* B:2 JRMT12. This study primarily aims to investigate the potential of *P. multocida* B:2 GDH7 strain as a delivery vehicle for DNA vaccine for future multivalent applications.

**Results:**

An investigation on the adherence, invasion and intracellular survival of bacterial strains within the bovine aortic endothelial cell line (BAEC) were carried out. The potential vaccine strain, *P. multocida* B:2 GDH7, was significantly better (*p* ≤ 0.05) at adhering to and invading BAEC compared to its parent strain and to *P. multocida* B:2 JRMT12 and survived intracellularly 7 h post treatment, with a steady decline over time. A dual reporter plasmid, pSRGM, which enabled tracking of bacterial movement from the extracellular environment into the intracellular compartment of the mammalian cells, was subsequently transformed into *P. multocida* B:2 GDH7. Intracellular trafficking of the vaccine strain, *P. multocida* B:2 GDH7 was subsequently visualized by tracking the reporter proteins via confocal laser scanning microscopy (CLSM).

**Conclusions:**

The ability of *P. multocida* B:2 GDH7 to model bactofection represents a possibility for this vaccine strain to be used as a delivery vehicle for DNA vaccine for future multivalent protection in cattle and buffaloes.

## Background

Haemorrhagic septicemia (HS) is a major disease in cattle and buffaloes caused by the infection of *Pasteurella multocida* [1]. In Asia, *P. multocida* B:2 is the serotype responsible for the disease [2]. Transmission occurs from diseased animals or carriers by means of intranasal and oral routes [1]. Invasion of the bacteria through endothelial cells result in rapid infiltration of the animal bloodstream [3]. Vaccination against HS is usually conducted prior to rainy seasons using oil-adjuvant vaccines or alum-precipitated vaccines [4]; however both bacterin vaccines only confer short-term protection. Commonly used live attenuated vaccines against HS consist of live organisms, such as the attenuated bacteria with reduced virulence compared to the wild-type [5]. *P. multocida* B:2 GDH7 is an attenuated derivative of the wild-type *P. multocida* B:2 isolated from a previous outbreak in Malaysia, that upon intranasal administration is an efficient vaccine for HS [6]. This strain was genetically modified by the disruption of the wild-type *gdhA* gene with the insertion of a kanamycin cassette [7]. This resulted in an interference of bacterial metabolism hence arresting its pathogenicity. Subsequently, it was also reported that a mutant strain known as *P. multocida* B:2 JRMT12, derived from a parent strain of Sri Lanka origin, *P. multocida* B:2 85,020 was developed and can be administered intramuscularly to confer a high degree of protection as a live vaccine in a mouse model of HS [2]. Since alum precipitated vaccine and oil-adjuvant vaccines are less effective against HS, an alternative is therefore crucially needed. The aforementioned mutants (*P. multocida* B:2 GDH7 and *P. multocida* B:2 JRMT12) have been found to be good candidates for attenuated *P. multocida* B:2 vaccine development in vivo [2, 8]. In this study, the interaction rate of both attenuated vaccine strains, *P. multocida* B:2 GDH7 and *P. multocida* B:2 JRMT12 towards bovine aortic endothelial cells (BAEC) was assessed. Moreover, the ability and efficiency of *P. multocida* B:2 GDH7 to persist in the intracellular environment of the host cells and to transfer plasmid DNA intracellularly was investigated. To assess this interaction, a dual-reporter plasmid that expresses in both prokaryotic and eukaryotic cells was used. It is crucial to understand the bacterial pathogenesis during progression of this disease particularly towards the fate of the plasmid carried by the bacterium after it enters into mammalian cells to further strengthen the ability of *P. multocida* B:2 GDH7 as a vaccine.

## Methods

### Bacterial strains and growth condition

Bacterial strains used in this study were: *P.multocida* B:2 wild-type, a local isolate from a previous outbreak of haemorrhagic septicaemia in Malaysian cattle, *P.multocida* B:2 GDH7, ∆*gdhA* derivative *P.multocida* B:2 wild-type and *P. multocida* B:2 JRMT12, an *∆aroA* mutant of strain *P. multocida* B:2 85,020 from an outbreak in Sri Lanka. In previous studies, stability test for both mutant strains *P.multocida* B:2 GDH7 and *P. multocida* B:2 JRMT12 have been reported [2, 7]. To determine the stability of all bacterial strains, each strain was passaged several times and growth studies were conducted prior to the interaction assays.

The bacterial strains are classified as biosafety level 2. All strains were cultured using Brain Heart Infusion (BHI) agar and broth (Oxoid) at 37 °C and shaken at 180 rpm. Whenever required, a total concentration of 50 μg/ml kanamycin and 60 μg/ml of streptomycin were added.

### Preparation of bovine aortic endothelial cell (BAEC)

BAEC (Cells applications. Inc., catalogue no. B304–05) was cultured in Dulbecco’s Modified Eagle’s Medium (DMEM 08459, Nacalai Tesque) supplemented with 10% fetal bovine serum (FBS, I-DNA), 1% glutamine and antibiotics (100 μg/ml of streptomycin and 100 U/ml of penicillin). BAEC was passaged accordingly and was maintained in complete DMEM medium with incubation at humidified environment of 5% (*v*/v) CO_2_ and 95% (*v*/v) air at 37 °C. All experimental assays were performed at the third cell passage.

### BAEC cell viability assessment

Cell viability was assessed using the trypan blue exclusion method [9]. A mixture (1:1) of the cell suspension with trypan blue (0.4% *w*/*v*) was placed in an improved Neubauer slide (1/400 mm^2^ × 0.1 mm depth). The slide was viewed under a light microscope, where viable cells will confer a clear cytoplasm whereas nonviable cells will be stained blue.

### Adherence assay

All *Pasteurellaceae* strains were harvested from 18 h cultures to achieve the optimum multiplicity of infection MOI (100 bacteria/mammalian cell). The washing step was performed twice using phosphate-buffered saline (PBS) before was resuspension of bacterial pellet in DMEM without antibiotics. Trypan blue exclusion assay was performed to monitor the concentration of BAEC in each well of a 24-well tissue culture plate before addition of the appropriate amount of bacterial suspension. The plate was then centrifuged at 300 x g for 5 min using an Eppendorf 5430 R centrifuge to disperse the bacteria onto each cell. The plate was then incubated for 2 h at 37 °C supplemented with 5% (*v*/v) CO_2._ Loosely bound bacteria were washed twice using PBS prior to trypsinization with 1 ml of 0.5% (*w*/*v*) trypsin-EDTA (pH 7.0) per well and incubated for 5 min at 37 °C with 5% (*v*/v) CO_2_. BAEC were harvested into the centrifuge tubes using PBS and the remaining cells in the plate were recovered using PBS. The suspensions were then centrifuged at room temperature for 5 min. In order to remove any remaining bacteria and any traces of trypsin-EDTA, the pellets were washed once using PBS before suspended in DMEM without antibiotics. An aliquot of cells were removed from each tubes to assess cell viability. By adding digitonin, the remaining cells was lysed during incubation at 37 °C in 5% (*v*/v) CO_2_ for 30 min. The cell suspensions were serially diluted and plated onto BHI agar followed by overnight incubation at 37 °C. Adhesion is expressed as the average value of bacteria/BAEC after incubation of bacteria with BAEC at MOI of 100:1 for 2 h. Data represent the means (±SEM) of three independent assays with duplicate samples.

### Invasion assay

The invasion assay was conducted according to the adherence assay with an additional incubation step. After 2 h incubation of the bacteria/BAEC cell mixture in the 24-well plate, the wells were washed twice with PBS to remove loosely bound bacteria. Approximately 1 mL of DMEM with polymyxin B (50 μg/mL) and gentamicin (50 μg/mL) was added in each well to eliminate the remaining extracellular bacteria. The plate was then incubated for 1 h at 37 °C in 5% (*v*/v) CO_2_. BAEC cell viability assessment were performed according to Section 2.3. Invasion rate was expressed as the average value of bacteria/BAEC that survive after 1 h exposure of polymyxin B and gentamicin with each final concentration of 50 μg/mL after 2 h infection at MOI 100:1. The data represented as the means (± SEM) of three independent assays with duplicated samples.

### Intracellular survival assay

Invasion assays were performed towards all three strains with an additional incubation period after the antibiotic treatment step, and before the viable cells were counted. In order to determine the potential intracellular growth of all strains, the number of viable BAEC was counted at various stages. The mixture of bacteria and BAEC were further incubated up to 4 h after the standard invasion assay without the presence of antibiotics, polymyxin B and gentamicin (P&G) at different concentrations of either 10 mg/ml or 50 mg/ml. These are to avoid the risk of any remaining extracellular bacteria to replicate after the initial antibiotic treatment without the presence of P&G. The method for assessment of BAEC cell viability was conducted according to the invasion assay above.

### Construction of dual-reporter plasmid, pSRGM

**Fig. 1:**
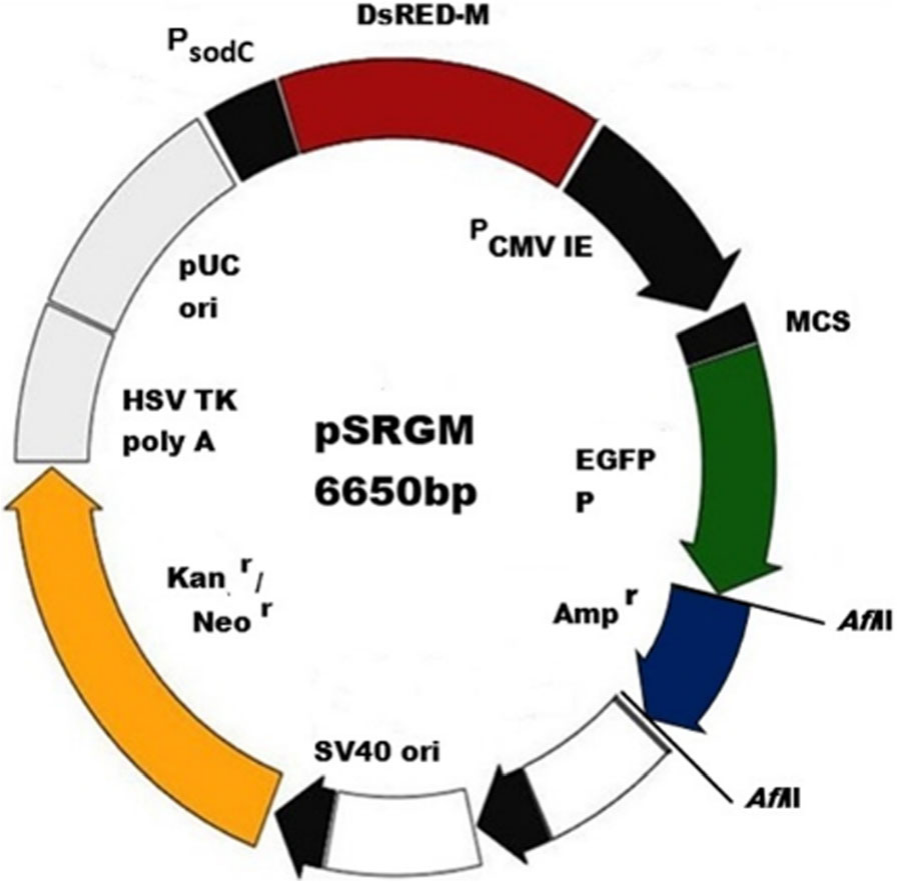
Plasmid pSRGM with two expression systems, a red reporter system that functions in prokaryotic cells driven by the ^*P*^sodC promoter and a green reporter system that functions in eukaryotic cells driven by the ^*P*^CMV_IE_ promoter

A plasmid to track the location and viability of bacterial cells when moving from extracellular into intracellular compartment of mammalian cells, known as pSRG was previously developed by Othman et al. [5]. In this study, pSRG was slightly modified to enable selection in *P. multocida* B:2 GDH7. Previously, *P. multocida* B:2 GDH7 was constructed by the insertion of a kanamycin cassette into one of the housekeeping genes to confer resistance to kanamycin [7]. Similar to *P. multocida* B:2 GDH7, pSRG is also resistance towards kanamycin. Therefore, the ampicillin cassette (amplified by PCR from pDSRed-Monomer, Clonetech, USA) was inserted into pSRG at *Afl*II sites and used for antibiotic selection of the plasmid (Fig. 1). The modified plasmid, pSRGM was then electroporated into *P. multocida* B:2 GDH7.

### Intracellular trafficking of *P. multocida* B:2 GDH7 pSRGM within BAEC

BAEC were prepared similar to the invasion assay. The only difference on preparation of slides for confocal laser scanning microscopy (CLSM) was that the BAEC were seeded in a removable chamber-slide (SPL Life Sciences, Korea) rather than a 24-well plate. The reason for that because when the well was removed from the chamber slide, the cells can be fixed directly onto the slide. The monolayer was washed twice with PBS after the antibiotic treatment. Approximately 200 μl of pre-warmed 4% (*v*/v) paraformaldehyde (PFA) was then added to each well and incubated at 37 °C with 5% (*v*/v) CO_2_ for 30 min. The cells were washed three times in order to remove PFA. An aliquot of HCS CellMask™ blue stain (Life technologies, USA) (5 mg/ml) was added to each well to counterstain the cells. The chamber-slide was then incubated for 7 min at same conditions as PFA. The staining solution was removed from the chamber-slide and washed three times with PBS. The wells were carefully removed from the chamber-slide and the slide was left to dry. Prolong gold antifade reagent (Life technologies, USA) was used as a mounting medium, followed by the coverslip. Slides were then labelled and stored in the dark until viewing.

### Confocal laser scanning microscopy (CLSM)

Fluorescence imaging was done with a Leica TCS SP5 II microscope (Leica microsystem, Germany) that was connected to Las AF software to capture images. The system allows visualization of fluorescence at resolution (1024 × 1024 pixels) and bit depth of 8-bit gray scale. The images were then processed with Leica application suite X (Las X) software and the 3D images were processed using LAS X 3D visualization software.

### Statistical data

In this study, Microsoft excel was used for calculating mean values, standard deviations and standard errors. For adherence, invasion and intracellular survival assay, student’s t-test and one-way ANOVA were applied.

## Results

### Cytotoxicity effects of *P. multocida* B:2 on viability of BAEC

**Fig. 2:**
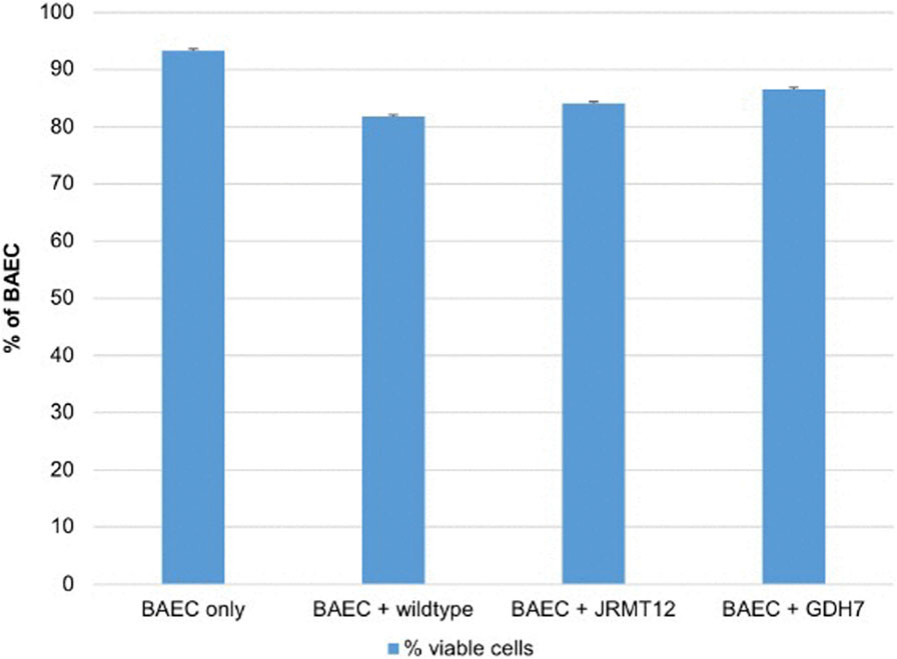
Percentage of viable and non-viable BAEC at 3 h post-invasion. Data represent the means (±SEM) of three independent assays with duplicate samples

In this experiment, the cytotoxicity effect of *P. multocida* B:2 wild-type as as well as two attenuated strains, *P. multocida* B:2 GDH7 and *P. multocida* B:2 JRMT12 towards the viability of BAEC were determined. Figure 2 showed that all bacterial strains possessed no cytotoxicity effects towards the BAEC at 3 h post-infection. All control and experimental cells were found to be at least 80% viable at the end of the assay.

### Adherence of *Pasteurellaceae* strains to BAEC

The ability of *Pasteurellaceae* strains to adhere to BAEC were investigated and compared in the adherence assay. Using one-way ANOVA test, *P. multocida* B:2 strain GDH7 showed significantly (*p* < 0.05) higher adherence to BAEC (27.46 ± 1.14 bacteria/BAEC) compared to *P. multocida* B:2 wild-type and *P. multocida* B:2 strain JRMT12 (12.30 ± 1.34 and 17.57 ± 1.32 bacteria/BAEC, respectively) (Table 1).

**Table 1:** Comparison of adherence rate of BAEC by *Pasteurellaceae* strains

Bacterial strains	Bacterial adhesion (No. of bacteria/BAEC)
*Pasteurella multocida* B:2 wild-type	12.30 ± 1.34*
*Pasteurella multocida* B:2 GDH7	27.46 ± 1.14*
*Pasteurella multocida* B:2 JRMT12	17.57 ± 1.32*

Adhesion is expressed as (no. of bacteria/BAEC) that has been deducted from the internalized bacteria by bacterial count after overnight incubation. Data represent the means (±SEM) of three independent assays with duplicate samples

* Signify significant differences (*p* < 0.05) with other strain

### Invasion rate of *Pasteurellaceae* strains towards BAEC

The adherence assay showed that all three strains were able to adhere to BAEC. Similarly shown in Table 2, all strains were detected intracellularly during the invasion assay. *P. multocida* B:2 GDH7 showed the highest invasion rate of 3.12 ± 0.06, followed by the wild-type and Sri Lankan strains (1.43 ± 0.17 and 1.38 ± 0.05 bacteria/BAEC, respectively). These findings suggested that the bovine *Pasteurellaceae* strains were able to adhere to and persist intracellularly in BAEC.

**Table 2:** Invasion rate of the *Pasteurellaceae* strains towards BAEC at MOI 100:1

Bacterial strains	Bacterial invasion (No. of bacteria/BAEC)
*Pasteurella multocida* B:2 wild-type	1.43 ± 0.17*
*Pasteurella multocida* B:2 GDH7	3.12 ± 0.06*
*Pasteurella multocida* B:2 JRMT12	1.38 ± 0.05*

Invasion is expressed as no. of bacteria/BAEC that resisted exposure to polymyxin B and gentamicin at a final concentration of 50 μg/ml for 1 h after an infection period of 2 h. Data represent the means (±SEM) of three independent assays with duplicate samples

* Signify significant differences (*p* < 0.05) with other strain

### Intracellular survival of the *Pasteurellaceae* strains in BAEC

The number of viable intracellular bacteria in BAEC was determined in order to demonstrate intracellular bacteria survivability, or the potential of intracellular replication of the bacterial strains within the cytoplasmic compartment of BAEC. Table 3 indicates that all three *Pasteurellaceae* strains at three different conditions showed a consistent decline in the number of viable intracellular bacteria per cell. Moreover, the pattern across all strains were considered not significant (*p* > 0.05). This may be attributed by an inability of the intracellular bacteria to replicate within the cell and is gradually eradicated by the mammalian cells with time.

**Table 3:** Intracellular survival of *P. multocida* B:2 GDH7, *P. multocida* B:2 JRMT12 and *P. multocida* B:2 wild-type in BAEC at MOI 100:1

		No. of viable intracellular bacteria/BAEC			
Bacterial strain	Invasion time (H)	Standard invasion assay	No antibiotic added after washing	P&G 10 μg/ml added after washing	P&G 50 μg/ml added after washing
*P. multocida* B:2 GDH7	3	3.00 ± 0.13	-	-	-
	5	-	2.62 ± 0.12	1.87 ± 0.17	1.90 ± 0.06
	7	-	1.73 ± 0.04	1.61 ± 0.04	1.59 ± 0.05
*P. multocida* B:2 JRMT12	3	1.60 ± 0.09	-	-	-
	5	-	0.93 ± 0.03	0.82 ± 0.06	0.67 ± 0.05
	7	-	0.78 ± 0.03	0.75 ± 0.01	0.63 ± 0.01
*P. multocida* B:2 Wild type	3	1.22 ± 0.21	-	-	-
	5	-	1.11 ± 0.04	0.94 ± 0.01	0.71 ± 0.04
	7	-	0.93 ± 0.01	0.83 ± 0.01	0.78 ± 0.03

Internalized viable bacteria were evaluated as no. of bacteria/BAEC after incubation of BAEC with bacteria for 2 h followed by exposure to polymyxin B and gentamicin (P&G) each at a final concentration of 50 μg/ml for 1 h prior to further incubation for 2 and 4 h in the presence of either 0, 10 or 50 μg/ml each of polymyxin B and gentamicin in the medium. Data represent the means (±SEM) of three independent assays with duplicate samples

### Intracellular trafficking of *P. multocida* B:2 GDH7 pSRGM within BAEC

**Fig. 3:**
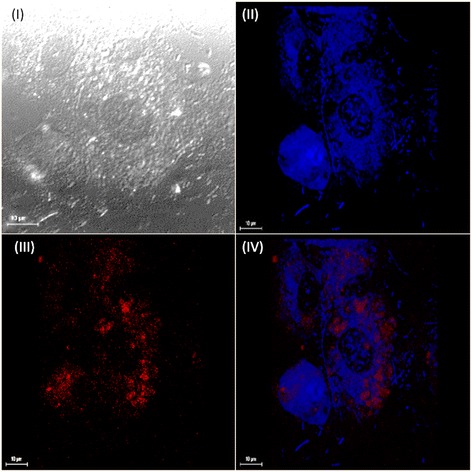
Localization of prokaryotic protein expression. Images showed BAEC with *P. multocida* B:2 GDH7 pSRGM at 3 h post-invasion. Cells were viewed under 60× objective lens with a Leica confocal laser scanning microscope (CLSM). Images were taken of the same field under three different light paths; (*I*): Image taken under DIC (*white*) light. (*II*): Image taken under filtered fluorescent light (Exc. = 415 nm, Ems. = 493 nm) (*blue*) indicating the plasma membrane of BAEC. (*III*): Image taken under filtered fluorescent light (Exc. = 570 nm, Ems. = 700 nm) (*red*) which are referring to *P. multocida* B:2 GDH7. (*IV*): Overlay of image (*II*) and (*III*)

**Fig. 4:**
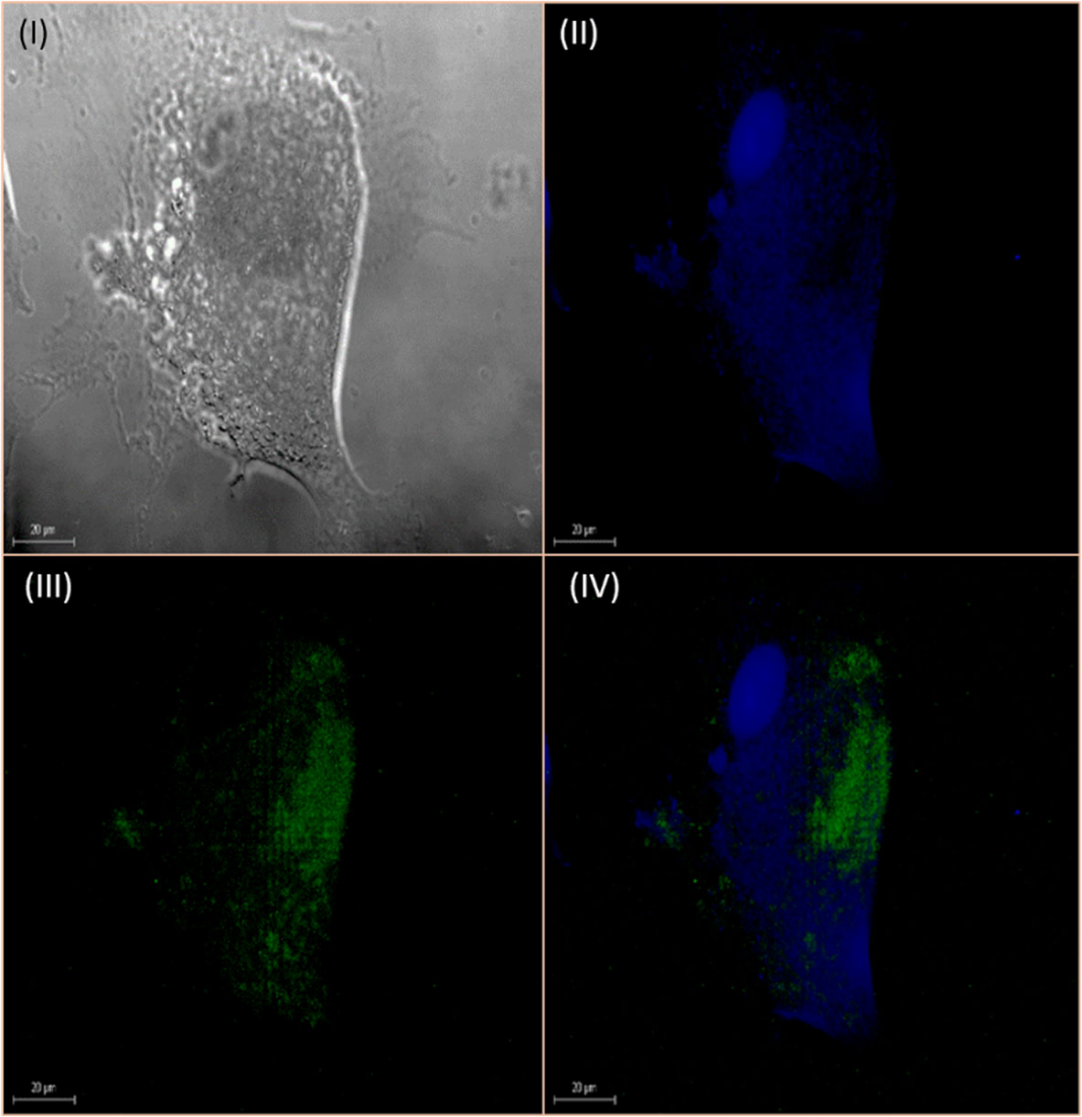
Localization of eukaryotic protein expression. Images showed BAEC with *P. multocida* B:2 GDH7 pSRGM at 5 h post-invasion. Cells were viewed under 60× objective lens with a Leica confocal laser scanning microscope. Images were taken of the same field under three different light paths; (*I*): Image taken under DIC (*white*) light. (*II*): Image taken under filtered fluorescent light (Exc. = 415 nm, Ems. = 493 nm) (*blue*) indicating the plasma membrane of BAEC. (*III*): Image taken under filtered fluorescent light (Exc. = 498 nm, Ems. = 540 nm) (*green*) indicating that the plasmid has been released into BAEC cytoplasm. (*IV*): Overlay of image (*II*) and 4.13 (*III*)

**Fig. 5:**
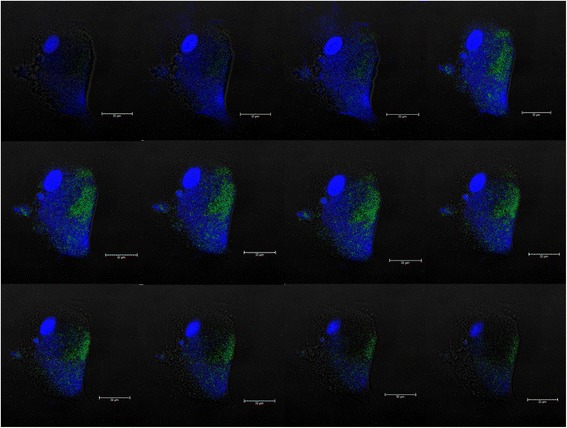
Z-stack images on GFP expression during bactofection. Optical sectioning of BAEC at 1.73 μm intervals confirmed that the cell was expressing GFP at 5 h post-invasion. The images were arranged accordingly from *top* to *bottom* view (*left to right*)

The functionality of each expression cassettes in pSRGM was individually assessed. Both prokaryotic and eukaryotic expression cassettes were shown to independently express the reporter proteins in their respective hosts (data not shown). In order to visualize the intracellular trafficking of *P. multocida* B:2 GDH7 pSRGM within BAEC, both the mammalian and bacteria cells were prepared as previously described in Section 2.4. Slides shown in Figs. 3, 4 and 5 were viewed under the 60× objective lens of the CLSM. Images from different light paths were captured at the same field of the slides. Images from Fig. 3 were obtained from slides prepared during 3 h invasion time. *P. multocida* B:2 GDH7 pSRGM (red) shown some internalization into BAEC, while no expression of GFP was detected. Therefore, this indicated that the plasmid was not released by the bacteria into the intracellular compartment of BAEC 3 h post-invasion.

Figure 4 showed a single BAEC expressing GFP within the cell membrane. This data was subsequently corroborated with an optical sectioning of the sample at 1.73 μm intervals from top to bottom with a total section stacking of 88.23 μm, as shown in Fig. 5. From these images, intracellular bacteria was found to be lysed and undetectable 5 h post-invasion. However, plasmid pSRGM was found to be released into the BAEC cytoplasm from the expression of GFP by the cells.

## Discussion

Live-attenuated vaccines confer the advantage of mimicking early stages of the natural route for infection. Intranasal administrations not only stimulates the mucosal immunity of the exposed host, but also transmit the organism to the in-contact host to stimulate mucosal immunity. Rafidah et al. [8] proved that intranasal (i.n.) inoculation with *P. multocida* B:2 GDH7 induced strong immunity in exposed and in-contact buffaloes that protected the buffaloes against wild-type *P. multocida* B:2 challenge. This may be pivotal in overcoming difficulties faced with current vaccines, such as the difficulty in gathering free-ranging animals for vaccination [10].

In this study, the attenuated derivative *P. multocida* B:2 GDH7 strain was shown to adhere to BAEC significantly better (*p* < 0.05) than its parent strain and *P. multocida* B:2 JRMT12 [2]. This is important especially in the development of a potential vaccine strain as a delivery vehicle for DNA vaccine. A higher number of viable intracellular bacteria will increase the chances of higher accumulation of DNA in vivo. The significant differences between the two potential vaccine strains was most probably due to the different mutations introduced in the *aroA* gene of the JRMT12 and the *gdhA* gene of the GDH7 derivatives. Mutation of *gdhA* gene of the GDH7 derivative renders an inability of this strain to convert α-ketoglutarate to glutamate [11] involved in the amino acid transfer in the bacteria. Hence, the bacteria has limited access to glutamate in the host which restricts bacterial replication before clearance from the host**.**

Assessment of the pathogen movement to the vascular system through the epithelial tissues is one of the important stages in pathogenesis of infectious *P. multocida* B:2. To be able to invade the bloodstream efficiently, cells that translocate through the blood vessel by adhering and invading may have a higher potential to model this mechanism. Previously, Othman et al. [12] demonstrated the capacity of the Sri Lankan *P. multocida* B:2 strains to be internalized and to persist up to 7 h in embryonic bovine lung cells. Similarly in this study, the Malaysian *P. multocida* B:2 strain and its derivative was internalized into BAEC 3 h post-infection and persisted up to 7 h post-infection, although a stable degeneration in viability with time was observed. During cytotoxicity assessment by trypan blue exclusion method, BAEC did not show any cytotoxicity effects towards any bacterial strains that were tested. Similarly, Galdiero et al. [3] evaluated cytotoxicity effects by quantifying the amount of lactic dehydrogenase released from BAEC after infection with *P. multocida* serotype B. The strain was reported to have almost no cytotoxicity towards BAEC.

The capacity of this vaccine strain to invade shows a promise to be developed as a candidate of a bactofection-derived system. This is an alternate approach to express plasmid-encoded heterologous proteins in a large subset of cells. However, not all attenuated bacterial strains have the same pattern to deliver foreign antigens. For example, in *Shigella spp.* and *L. monocytogenes* exogenous DNA was lysed after delivery to the cytosol of infected hosts [13, 14]. Other findings that described localisation of released DNA in the nuclei and phagosomal compartments of infected hosts of *Salmonella*, *Yersinia* and *E. coli* occurred to mechanisms that are unknown [13, 15, 16]. According to Othman et al. [5], bactofection using an attenuated *P. multocida* B:2 JRMT12 pSRG towards embryonic bovine lung cell (EBL) showed plasmid localisation in the nuclear compartment of the cells from the vacuoles through the cytoplasm. This corroborated with data obtained from this study, where bacterial RFP expression were observed intracellularly 3 h post-invasion (Fig. 3). This also suggested that the bacterial cell was not lysed, and the plasmid remained within the bacterial cytoplasm. However Othman et al. [5] reported that as early as 3 h post-infection, some of the EBL cells managed to express GFP and may indicate that the bacteria was lysed, leading to a transfer of the plasmid into the host cytoplasm. It was also observed that bacterial lysis and the released of plasmid into the host cytoplasm occurred only 5 h post invasion, as shown in Figs. 4 and 5. In this study, the internalized bacteria remained viable and detectable from 3 to 7 h after infection in adherence assays, but however was difficult to locate 5 h post-infection in the bactofection assay.

## Conclusions

In conclusion, the capacity of *P. multocida* B:2 strains to be internalized and persist up to 7 h in mammalian cell was demonstrated. However, this study shows that the local attenuated strain, *P. multocida* B:2 GDH7 were significantly more efficient in internalizing within cells as compared to its parent strain and the Sri Lankan attenuated strain, *P. multocida* B:2 JRMT12. Additionally, not only does the Malaysian attenuated strain, *P. multocida* B:2 GDH7 efficiently vaccinated via the intranasal routes [17] but this study showed that the vaccine strain is a promising candidate as a delivery vehicle for DNA vaccine (bactofection). In the future, both reporter proteins in the pSRGM can be replaced with other antigenic genes from different bovine diseases in order to promote multivalent protection against cattle diseases. It will also be interesting to further investigate the transfer mechanism of plasmid in vivo from the bacteria to the host cells. This could provide evidence towards enhancement of the efficacy of DNA vaccine delivery via bactofection.

## Abbreviations

AP: Ampicillin
*aro*A: Aromatic amino acid metabolism gene
B:2: *Pasteurella multocida* biotype B capsular serotype 2
BAEC: Bovine Aortic Endothelial Cell
BHI: Brain Heart Infusion
CD: Cytochalasin D
CFU: Colony forming unit
DNA: Deoxyribonucleic acid
EBL: Embryonic Bovine Lung
EMS: Emission
Exc: Excitation
FBS: Fetal Bovine Serum
FI: Fluorescence intensity
*gdh*A: Glutamate dehydrogenase gene
GFP: Green fluorescent protein
GM: Gentamicin
HS: Haemorrhagic septicaemia
i.m: Intramuscular
i.n.: Intranasal
MOI: Multiplicity of infection
Ng: Nanogram
PBS: Phosphate buffer saline
PCR: Polymerase chain reaction
PFA: Paraformaldehyde
RFP: Red fluorescent protein
rpm: Revolutions per minute
Sm: Streptomycin
TAE: Tris acetate EDTA
UV: Ultraviolet
